# A Novel Hybrid Convolutional Neural Network Approach for the Stomach Intestinal Early Detection Cancer Subtype Classification

**DOI:** 10.1155/2022/7325064

**Published:** 2022-06-24

**Authors:** Md Ezaz Ahmed

**Affiliations:** College of Computing and Informatics, Saudi Electronic University, Riyadh, Saudi Arabia

## Abstract

There may be different types of cancer that cause fatal effects in the human body. In general, cancer is nothing but the unnatural growth of blood cells in different parts of the body and is named accordingly. It may be skin cancer, breast cancer, uterus cancer, intestinal cancer, stomach cancer, etc. However, every type of cancer consists of unwanted blood cells which cause issues in the body starting from the minor to death. Cancer cells have the common features in them, and these common features we have used in our work for the processing. Cancer has a significant death rate; however, it is frequently curable with simple surgery if detected in its early stages. A quick and correct diagnosis may be extremely beneficial to both doctors and patients. In several medical domains, the latest deep-learning-based model's performance is comparable to or even exceeds that of human specialists. We have proposed a novel methodology based on a convolutional neural network that may be used for almost all types of cancer detection. We have collected different datasets of different types of common cancer from different sources and used 90% of the sample data for the training purpose, then we reduced it by 10%, and an 80% image set was used for the validation of the model. After that for testing purposes, we fed a sample dataset and obtain the results. The final output clearly shows that the proposed model outperforms the previous model when we compared our methodology with the existing work.

## 1. Introduction

Cancer is a type of body unwanted blood cells that is considered to be the second most frequent occurring disease globally [1–3]. A virus known as the human papillomavirus (HPV) is the cause of body cancer. The virus has the ability to harm cells in the body. The dysplasia cells resemble cancer cells, yet they are not cancerous. Common Intraepithelial Neoplasia (CIN) is the name given to these cells. CIN is divided into three classes based on severity viz. CIN1, CIN2, and CIN3. CIN1 will relapse over time due to the body's immunological system. As a result, the primary objective is to identify CIN2 and CIN3. If the proper sort of body is recognized early on, the treatment will be shown beneficial. Early identification of cancer can boost the five-year survival rate to 91 percent, according to Cancer Statistics [4–6]. Cancer screening procedures that are routinely utilised include the Pap test, HPV testing, colposcopy, and digital angiography. These are useful screening procedures; however, they have limited sensitivity in identifying CIN2/3+. Furthermore, these examinations necessitate the use of skilled staff and a laboratory setting. Because the entire abovementioned screening test includes human intervention, it is more vulnerable to human mistakes [7–10]. Deep learning and computer vision have been shown to be useful in the healthcare area for medical image categorization. The experimentation in this paper is based on a picture dataset that is accessible through some public open data sources. In June 2017, Kaggle issued a challenge by making thousands of body datasets available to the public dataset via the Intel and Mobile ODT competition. The assignment involved categorising one's body image into one of three categories. We have suggested a novel algorithm to solve the aforementioned problem [11–15].

There may be different types of cancer present in the body. Cancer is the unwanted occurrence of blood cells in different parts of the body [16–25]. According to the body parts, the blood cells grow unnaturally in categories such as skin cancer, intestine cancer, and uterus cancer. In women, it is a kind of cancer that affects the lower region of the uterus. It is connected to the uterus and the vaginal divider [26–30]. The body resembles a little doughnut with a small aperture in the centre. The posture of a woman's body changes throughout her life. The body is seen using a tool known as a speculum. By inserting the speculum into the vaginal canal, professional practitioners examine the body and make a prediction [31]. It is caused by a virus called human papillomavirus (HPV) [32–40]. This HPV is found in everyone. Some HPVs are not dangerous, while others might cause abnormal cell development in the body [41–44]. HPV-related illnesses spread gradually. It takes between 10 and 20 years to progress from the precancerous to the cancerous stage. Similarly, cervical malignant development can be avoided if a cervical illness is detected and treated in the premalignant growth stage. With early detection, the rate of survival can be enhanced [45–50]. As indicated, body cancer is broadly categorised into three types: Types I, Type II, and Type III which are the first stage, the second stage, and the last third stage [51–57], respectively. There is a fine boundary between these three types, making it difficult for healthcare personnel to diagnose. The work may get simpler as technology advances. In our work, we have gathered the dataset from different sources and considered our work for the common cancer cell diagnosis.

## 2. Related Work

The craniocaudal (CC) image and the mediolateral oblique (MLO) view of the intestines are supplied simultaneously during the analysis and diagnosis of any type of cancer screening. Mammograms obtained from many perspectives provide more information and characteristics about the lesion than a single scan [1–6]. As a result, mammography classification analysis approaches that are not confined to a single view but rather multiview are beginning to emerge. There are two categorization modes for multiview mammograms [7–10]. Binary segmentation masks or regions-of-interest (ROI) are required to engage in training in one modality. Authors in [11, 12] created a stomach classification method that combines two views of the same intestine and their respective lesion masks, in which each image and associated masks [13, 14] were trained separately, and then a final CNN classifier was trained with characteristics learned from both views. In [15], the authors have built an automatic analytic approach for multiview mammography categorization on this foundation. Using two views and their corresponding masks as input, authors in [16–19] have created a residual neural network to determine if a tumour is malignant or not. In [20],integrated ROIs from four images of both breast mammograms have been used for three-stage classification: abnormal or normal, calcification or tumour, and lastly benign or malignant. Papers [21–27] have suggested a model for obtaining multiscale classification features. The approaches described above still necessitate the use of extra masks or ROI annotations, which necessitate the expenditure of significant resources by a well-trained clinician. In the other mode, approaches for mammography classification analysis that do not require pixel-level or bounding box annotations are created. In [28], the authors have used four standard views of the right and left breasts to classify tests as Imaging Reporting and Data System (BI-RADS) 1, BI-RADS 2, and BI-RADS 3. Authors in [29, 30] have trained a CNN to distinguish between malignant and nonmalignant tumours using four standard views and heatmaps. In paper [31], authors have established a multiscale approach for cancer calcification classification that pulls information from each mammography image (CC and MLO views). For the multiview challenge, in research work [32–35], they created a convolutional neural network that joined four mammography pictures at the same time. Authors in [36] have introduced a two-stage approach for classifying two-view mammograms. A two-stage convolutional neural network model was used by the tools to classify benign and malignant mass cases. Based on the actual diagnosis process of the doctors combining the CC view and MLO view mammograms, the good performance brought by multiview methods benefits from complementary information between different views. On this basis, we consider both global and local features of mammograms to further improve the expressiveness of the model. An end-to-end neural network model that combines the CC and MLO views is trained only using image-level labels for the whole mammogram benign or malignant classification. Our model can effectively conduct global and local features in spatial dimensions to obtain a relatively more competitive result [37–40]. Many time series models still exploit simple regressive algorithms instead of deep learning [40–43]. Some reasons for this are interpretability, limited data, and low training cost. Attention mechanisms provide a compelling argument, and the results can be used to interpret the reasons for the performance improvements. In the healthcare domain, authors in [44, 45] have presented an interpretable bidirectional recurrent neural network-based approach (HAN-ECG) for the detection of Atrial Fibrillation (AF) based on the ECG recordings. It is a hierarchical attention network that provides three attention modules to realize multiresolution analysis in ECG leading to AF. One of the first papers [46] proposes to utilize 1-D convolution- and self-attention-based Simply Attend and Diagnose (SAnD) architecture with both single-task and multitask strategies for medical multivariate time series data. Since the self-attention mechanism examines relations for all pairs of timestamps, one of its biggest difficulties is the consideration of a long time series. To overcome this, a masking mechanism was used to hide timestamps that are too far in the past and the dense interpolation was applied instead of the addition and layer normalization components after the self-attention module. The authors in [47–53] have developed a novel temporal attention encoder-decoder model, called MTSMFF, for multivariate time series. The authors in [58] have used genetic algorithms for detection and prediction of cancer and critically analysed the state-of-the-art techniques used for cancer research. The authors in [59] have implemented a model with machine learning techniques such as support vector machine, logistic regression, and *K*-nearest neighbor (KNN) in breast cancer classification.

## 3. Proposed Methodology

**Figure 1:**
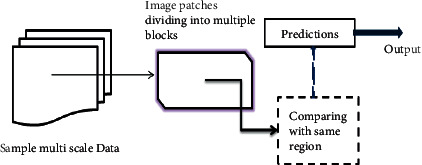
Process sampling dataset for multiscale module.

To judge the benign or malignant cancer cells by the whole mammogram or other radio processes, the multiview multifeatures convolutional neural model extracts feature information from the data input given to the convolutional model. The proposed network first extracts features separately through parallel convolution layers and fuses features, then obtains global context information from fused features through the self-attention mechanism, and finally refines important local features through multiplex convolutions.  Figure 1 represents the multifeatures extraction model architecture which mainly includes the following:

1. Pretrained ResNeXt: a few layers of the pretrained ResNeXt-101 with a 32 × 8*d* template are contributed to extracting whole blood cell features.
2. Transformer encoder: it extracts features to construct global features to overcome the limitation of the receptive field.
3. Multiplex convolutions: they send more refined local features to the classifier to get classification.
4. Classifier: it contains a fully connected layer and a softmax layer.

**Figure 2:**
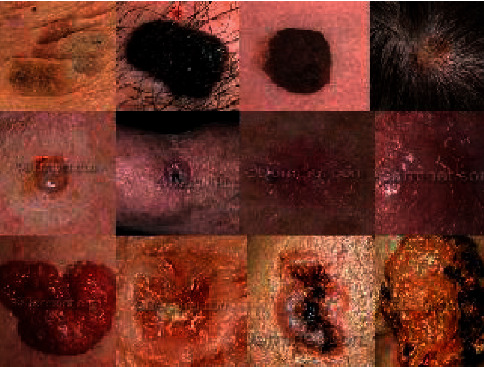
Representation of four subtype cancel cell images.

Figure 2 shows the processing of the sample data. We have shown some cancer blood cells of different types of cancer occurring in the body.

**Figure 3:**
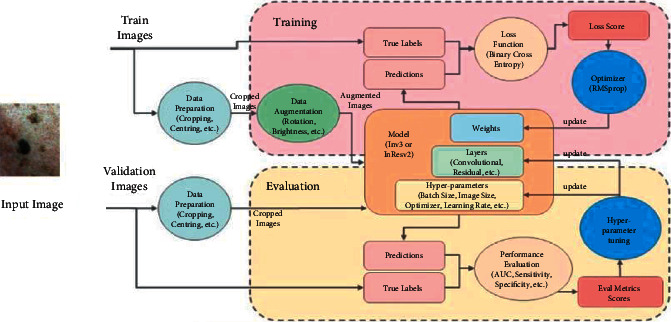
Input image undergoing training and validation process.

Different types of convolutions have different feature extraction capabilities. We propose a multiplex convolutions module to elaborate local features. Different from the standard convolution, the deformable convolution has an offset setting that allows the sampling grid to be deformed freely. The dilated convolution [47] is applied to convolution regions by a filter with a dilation factor. The two convolutions have powerful capabilities to extract features of multiple receptive fields from images while increasing the receptive field without subjoining the number of connections and calculation parameters.  Figure 3 shows the processing of the input images through the proposed model which passes through different stages to extract the multifeatures of blood cells and then a different dataset is processed for the validation of the model. Every type of cancer has a common lump of blood and distinguishes it from different body parts. To take full advantage of the characteristics, we gave proposed different layer convolutions, and we design three branches based on standard convolution, deformable convolution, and dilated convolution, respectively. We restore the sequence data from transformer layers into a standard feature map, which is then used as the input for each branch. In addition, global average pooling is concatenated after different convolutions to reduce the number of parameters and overfitting.

**Figure 4:**
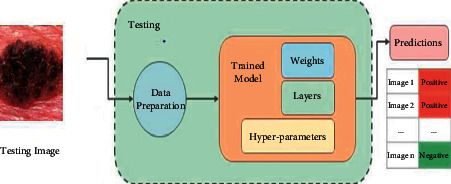
Testing process for the outcomes.

Figure 4 shows the testing of the model. The testing images go through different testing phases and give the output in terms of the classification of cancer cells, representing positive or negative.

The proposed method algorithm is shown as follows, and Table 1 represents the different feature cells extracted:

Table 1 gives a description of feature extraction with different types of filters.

ResNet introduces shortcut connections to form residual learning and used the gradient disappearance neural network method to solve the existing problem. Based on ResNet, ResNeXt which is a structural paradigm of split-transform merge replaces the standard convolution with grouped convolution, splits and merges the channel. Considering its advantages that improve the accuracy without increasing the complexity of the parameters and reducing the number of hyper parameters, we choose ResNeXt (ResNeXt with 101 layers and grouped convolution with 32 groups) as a base module to extract spatial features from the whole cancer cells. To save computational resources and reduce the number of parameters, instead of using the integrated ResNeXt-101 32 × 8*d* structure, we only use the first 7 × 7 265 convolution, max-pooling, and the first three convolution blocks as the first module of our model. Transfer learning reuses the weights of the pretrained model as the starting point for training our model, making the model robust and easier to train. Based on the idea of transfer learning, we choose the pretrained ResNeXt 32 × 8*d* to initialize our ResNeXt module. The pretrained model was first trained on the Instagram dataset that contains 9.4 × 108 images and 1,500 labels with weakly supervised learning and then fine-tuned on ImageNet [42]. The multiview and multifeatures extraction of cancer are denoted by Xcc and Xmlo. For each view, we apply the pretrained ResNeXt module for spatial feature extraction from the whole dataset and obtain a 1024-dimension hidden representation vector, respectively. In the proposed work, we have clearly trained the dataset and then processed it for the validation purpose from the CNN model; finally, the model is tested and obtains the output results. The proposed model outperforms the existing models.

## 4. Experimental Results

The model includes two subnetworks: a multiview CNN and a multidilated CNN, with the multiview CNN extracting features from multifeatures and feeding them into a multidilated CNN for classification. DLA-SE-Res2NeXt-60 [31] is a multiscale attention network model that introduced multiscale capability, the squeeze-and-excite method, and deep layer aggregation that harvests block- and layer-wise characteristics. MVNN [33] primarily combines a multiscale convolution module with an attention module for feature extraction.

The training set is made up of 90% of the samples in our dataset, whereas the testing set is made up of 35%. Only the training set, which is utilised to optimise our model parameters, is supplemented. Gradient descent with a stochastic component (SGD), the optimizer is used, with a momentum of 0.9 and a weight decay of 5.1%. A minibatch is defined as a group of eight sample data. The training epochs have been set to 100. The network with a factor of ten reductions in learning rate with an initial value of 0.001, 10 is added every 20 epochs. During the set, the number of transformer layers *L* = 12 in the transformer encoder module. We use Test Time during inference. The various mathematical parameters used are shown as follows:

(1) $$\begin{array}{c} T=\left\{\left(X_{1},Y_{1}\right),\left(X_{2},Y_{2}\right),\left(X_{3},Y_{3}\right),\ldots ,\left(X_{n},Y_{n}\right)\right\}. \end{array}$$

The following function shows the fitting curve function where *W* represents the weights of the layers and *b* is the bias factor introduced:

(2) $$\begin{array}{c} F\left(X\right)=W^{T}x+b. \end{array}$$

The loss function is given by

(3) $$\begin{array}{c} L\left(X,Y,F\right)=Y-F\left(X\right). \end{array}$$

The various mathematical factors used in the work are calculated using the standard formulas. Some of the mathematical equations are represented as follows:

(4) $$\begin{array}{c} AP=\sum_{k=1}^{n}\text{precision}\left(k\right)\times \Delta \text{recall}\left(k\right),\\ \text{Precision}=\frac{Ntp}{Ntp+Nfp},\\ \text{Recall}=\frac{Ntp}{Ntp+Nfn}, \end{array}$$

where *Ntp* is the number of correctly detected samples, *Nfp* is the number of falsely detected samples, and *Nfn* is the number of false samples.

Table 2 shows the classification results of the proposed CNN models utilised for classification purposes in comparison to certain sophisticated methods.

**Figure 5:**
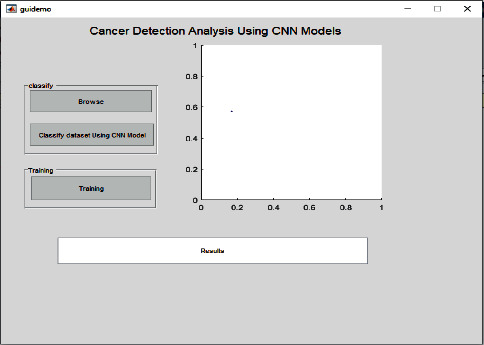
Cancer detection analysis template layout.

In addition, as shown in Figure 5, we present the base template and the simulation environment of our technique and the competing methods to visualise classification performance.

**Figure 6:**
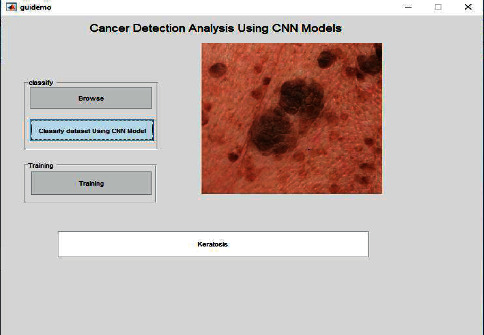
Cancer detected and classified after the testing phase.

Figure 6 shows the training processing of the dataset when fed to the model. It will undergo the training of the model and classification according to the process by convolutional neural network layers. The results will be shown in the result tab as well which is showing a kind of cancer detected and the type of cancer is keratosis. In the same manner, when the complete dataset is processed for the training of the model, it may show the positive and negative parameters.

**Figure 7:**
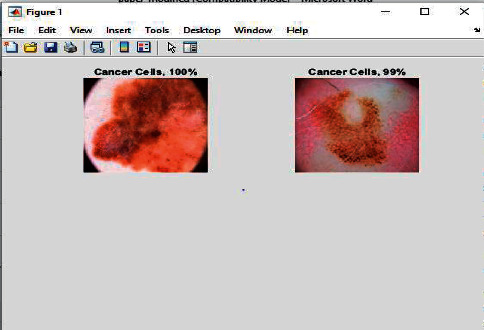
Cancer cells detected and shown the results with the % age of cancer cells.

In Figure 7, it has been shown that the input images come out to be detected as 100 percent cancer cell means it is true given the output as 1.

**Figure 8:**
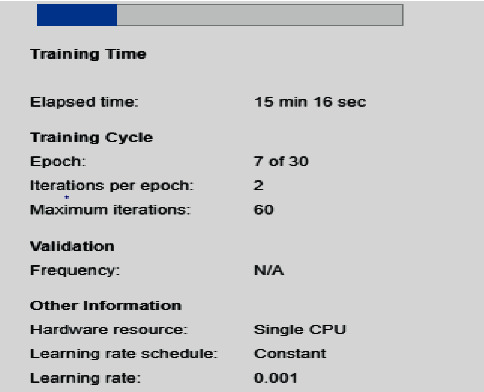
Intermittent processing of epoch/iteration.

Figure 8 shows the epoch processing time, as we are using the CPU of our laptop in the present work, which takes much time for the processing, so we stop the processing in between. The obtained result accuracy can be improved if the same model is used in a GPU environment.

**Figure 9:**
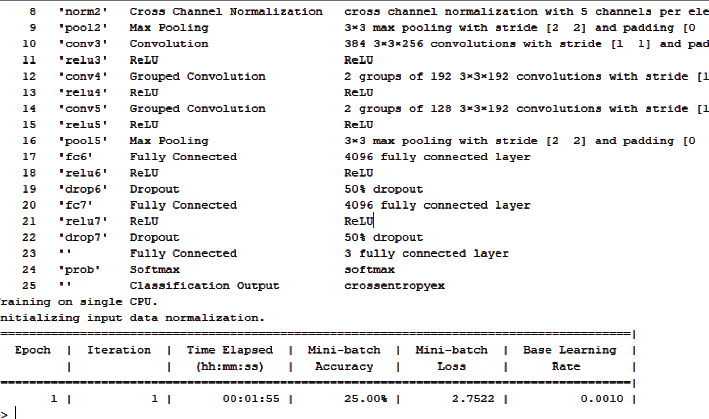
Convolutional output from different layers.

Figure 9 shows the output that comes out from the processing of different layers of the convolutional neural network. From iteration 1–7, the model takes for the initialization, and then from epoch 8 starts giving the output.

**Figure 10:**
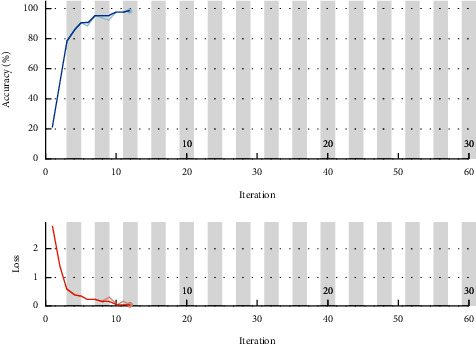
Performance analysis of accuracy (%) and loss function (%).

Figure 10 shows the processing analysis in terms of accuracy (%) and loss function. It clearly indicates that with the no. of iteration, the accuracy increases and the loss function decreases, which are an indication of the improved model.

**Figure 11:**
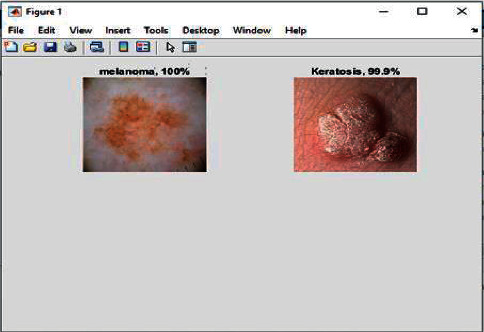
Cancer cells detected and classified.

**Figure 12:**
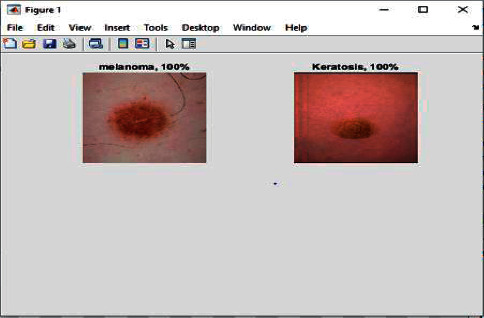
Cancer cells detected and classified.

Figures 11 and 12 show the different types of cancer cells detected and classified.

As already shown, Table 2 represents the calculated results from a different model and clearly indicates that the accuracy of the proposed model is much higher than the previous approaches along with better recall and precision values.

**Figure 13:**
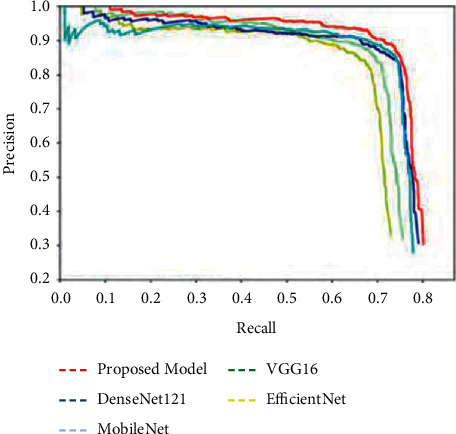
Analysis of precision with the recall values.

Finally, Figure 13 shows the graphical analysis of the precision with the recall values.

## 5. Conclusion and Future Scope

A multiconvolutional neural network learning approach is used in the proposed work to categorise entire cancer cell philosophy into normal and dangerous ones. Unlike the low-resolution images in the public dataset, the dataset used in our research work is a collection of samples collected in recent years using sophisticated technologies. The picture quality is assured, and it is more in accordance with contemporary clinical practice. For multiview mammography classification, we suggested a neural network based on global and local characteristics. Our model accepts both and shows better results in terms of accuracy, precision, and recall values. Given the limitations of the current study, we have indicated a few areas for future research. First, considering the importance of dataset size, we can investigate a larger dataset. Second, it is possible to explore integrating the annotations to improve accuracy. Third, multimodal analysis is an excellent way for any cancer screening and joint diagnosis integrating ultrasound and MRI which can be further analysed in future work.

## Figures and Tables

**Algorithm 1:**
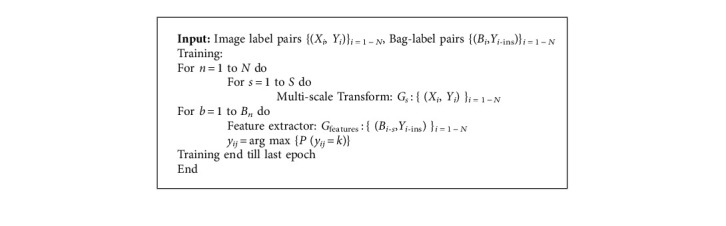
The Proposed Algorithm.

**Table 1:** Feature extraction.

	Types of filters	Description
Feature extractor	Canny	Used to detect and extract the features of the edges
	CR	Used to find the *L*_1_ regularized parameter value
	VLAD	Used to find the *k*-dimensional and *L*_2_ regularized parameter value

**Table 2:** Comparison of classification results of the proposed CNN model with sophisticated methods

Classification methods	Accuracy	Recall	Precision
DenseNet121	85.71	83.23	83.23
ResNet50	54.67	87.23	65.54
MobileNet	76.45	74.23	67.45
VGG16	83.45	48.34	73.56
MVNN	68.45	58.45	84.45
EfficientNet	46.53	65.45	45.67
Proposed method	**92.34**	**89.45**	**90.67**

## Data Availability

The data used to support the findings of this study are available from the corresponding author upon request.
